# A low-cost calcium silicate hydrate with an exceptionally large sequestration capacity for aqueous divalent nickel

**DOI:** 10.1038/s41598-026-51622-8

**Published:** 2026-07-07

**Authors:** Han Zhou, Pieter Bots, Bao Liu, Christopher Hall, Andrea Hamilton

**Affiliations:** 1 Department of Civil and Environmental Engineering, University of Strathclyde, 75 Montrose Street, Glasgow, G1 1XJ UK; 2 School of Engineering, University of Edinburgh, The King’s Buildings, Edinburgh, EH9 3JL UK; 3 Present Address: School of Civil Engineering, Nantong Institute of Technology, Nantong, China; 4 Present Address: National Defense College of Engineering, Army Engineering University of PLA, Nanjing, China

**Keywords:** Chemistry, Environmental sciences, Materials science

## Abstract

Nickel (Ni) is a high-value resource in industrial economies with essential uses in ferrous and non-ferrous alloys, electric-vehicle batteries, magnets, and catalysts, but has acute supply-chain risks and is classified as a critical material (or mineral). Here we show that large-scale spontaneous sequestration of nickel occurs in a low-cost calcium silicate hydrate material (CS) under mild conditions. CS takes up Ni^2+^ ions rapidly from aqueous solution to achieve loadings as high as 440 kg Ni per tonne CS. This chemistry brings new opportunities in nickel recycling, recovery and environmental clean-up. The Ni sequestration reaction of CS is similar to that previously described (A Hamilton et al., *Scientific Reports* (2024) 14:7052) for cobalt, although it is about four times faster. Full kinetic and reaction product data are reported. The reaction provides an easy synthesis route to a new Ni-phyllosilicate with a high metal loading, one of a class of materials in active development as catalysts for process-scale water-splitting and reforming reactions.

**Supplementary Information:**

The online version contains supplementary material available at 10.1038/s41598-026-51622-8.

## Introduction

Nickel [Ni] is a non-ferrous metal of the first economic importance with wide application across many industries – alloy steels, superalloys in jet engines, invars for LNG tankers, soft magnets in transformers, electrodes for EV and storage batteries, catalysts for green hydrogen production. World production of new nickel metal is about 3.5 Mt/y. Nickel is consistently classified as a critical material (or mineral) in major analyses^1–4^ of industrial strategy because of the supply-chain risk that arises from the dominance of Indonesia and the Philippines in mine production and of Indonesia and China in nickel refining. Nickel is one of six critical minerals for the global clean energy transition analysed for the G7 nations by the International Energy Agency^5^. IEA forecasts that demand for Ni doubles by 2050. Sustainability and circularity require a large increase in end-of-life recovery and recycling, especially in the battery sector, and also in recovery from mine waste^6^. Recovery from mine waste will be favoured by the gradual depletion of high-grade ores. New process streams such as methane reforming from CO$_{2}$^7^ and electrolytic hydrogen^8^ will encourage innovation in Ni catalysts. Sub-ppm limits on dissolved Ni concentrations in water imposed by WHO, EU and US EPA^9^ require effective methods of environmental clean-up^10^.

In a recent paper^11^ we described new chemistry for the sequestration of cobalt [Co] from solution using a calcium silicate hydrate available in the form of a low-cost commercial material [CS]. The CS “sponge” sequesters Co$^{2+}$ spontaneously at ambient temperature. The reaction is a stoichiometric 1:1 replacement of Ca by Co, and takes up as much as 440 kg Co per tonne CS, a loading hugely greater than can be achieved by adsorbents available today.

We now show that CS also removes nickel ions (Ni$^{2+}$) from aqueous solution in a similar way. As with Co, the reaction product is a poorly crystalline phyllosilicate. The sequestration reaction provides a way both to capture Ni$^{2+}$ ions for environmental clean-up and recycling, and also to produce a phyllosilicate precursor material with a high metal loading for application in catalysis^12^.

## Results and discussion

### CS characterization

The composition and structure of CS have been described previously^11^. In brief, CS, which is produced commercially by a hydrothermal process, consists largely of the calcium silicate hydrate mineral xonotlite Ca$_{6}\textrm{Si}_{6}\textrm{O}_{17}\text {(OH)}_{2}$ (Xon 91.3 wt%), with minor calcite (Cal 6.3 wt%) and fibrous cellulose (2.4 wt%) (see Supplementary Information). Only xonotlite takes part in the chemical reaction with aqueous Ni$^{2+}$ solutions. Xonotlite has a strongly fibrous habit^13^ with the fibre axis parallel to the silicate double chains that lie in the [010] crystallographic direction^14^. Double chains are formed by bridging-oxygen crosslinks shared between every third silicate tetrahedron of individual chains. Parallel double chains then lie between sheets of Ca polyhedra in the (001) crystallographic plane. OH groups are coordinated to the Ca$^{2+}$ ions. There is no molecular water in the xonotlite structure. While the general features of the crystal structure are well established, some complexity arises from the existence of a number of polytypes^15,16^. The polytypes describe the several ordered and disordered stacking arrangements that are possible between chains and sheets.

**Fig. 1:**
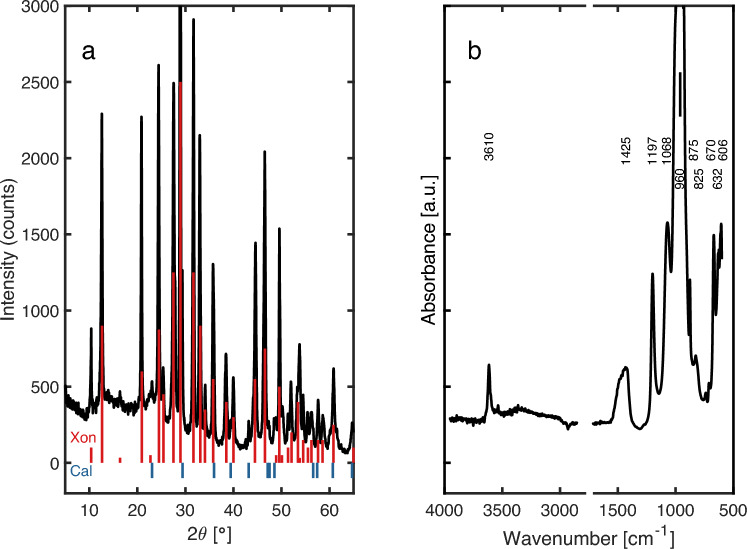
(**a**) Cu K*α * powder XRD pattern of CS at laboratory temperature: Xon, reference pattern of xonotlite from ICDD 23-125 with 102 and 500 reflections from COD 9009724^15^; Cal, reference pattern of calcite from ICSD 191852. (**b**) Mid-IR spectrum of CS at laboratory temperature: the complex overlapping bands in the region 600–1200 cm$^{-1}$ are due to vibrational modes of the silicate structure of xonotlite^17^. Weak bands at 1425 and 875  cm$^{-1}$ match reported $\nu _3$ and $\nu _2$ bands of calcite^18^.

The powder X-ray diffraction [XRD] pattern of the CS used (Fig. 1a) matches the reference pattern ICDD 23-125 of a xonotlite produced from lime and silica autoclaved at 200 $^{\circ}\textrm{C}$ for 24 h, apart only for the absence of the weak $\bar{1}$11 reflection at 18.4$^\circ$ *2θ *. While highly crystalline natural specimens of xonotlite can usually be described as mixtures of distinct ordered polytypes^16,19–21^ this is less feasible for synthetic xonotlites of poorer crystallinity. None of the reflections of CS in Fig. 1a are unique to individual polytypes.

The measured mid-IR spectrum of CS (Fig. 1b) is in good agreement with the published spectrum of a natural xonotlite mineral sample^17^. The intense band at 960 cm$^{-1}$ and the weaker one at 1068 cm$^{-1}$ are both assigned to Si-O-Si stretching vibrations of non-bridging tetrahedra^22^. The sharp band at 1197 cm$^{-1}$ is assigned^22,23^ to the Si-O-Si stretching mode of the crosslinks of the silicate double chain. The band at 1068 cm$^{-1}$ has also been attributed to an Si-O-Si stretching mode found only in double chain silicates^17,24^. The narrow band at 3611 cm$^{-1}$ is assigned to OH groups of xonotlite^17,25^. There is no evidence of molecular water in the region 3150–3650 cm$^{-1}$.

### Sequestration of Ni

#### Analysis of solutions

Sequestration of Ni by CS was studied by reacting powdered CS (passing 230 mesh sieve) with aqueous Ni$\text {(NO}_{3}\text {)}_{2}$ solutions of initial concentration $b({\textrm{Ni}})_0$ 0.339, 0.071, 0.034 and 0.007 *m* (where* m* denotes molal concentration: mol/kg water) for periods of 1, 2, 4, 6, 8, 15 h, 1:5, 12, 14, 28 and 56 days. For each test, 1.00 g of CS was mixed at room temperature with 25.0 mL aliquots of Ni solution or a control of deionised water. The aliquot with the highest solution concentration contained a little more Ni than needed to replace Ca entirely in 1.00 g CS. At each sampling time, the supernatant solution was analysed for Ni remaining in solution, and for Ca and Si released from the CS sequestrant.

**Fig. 2:**
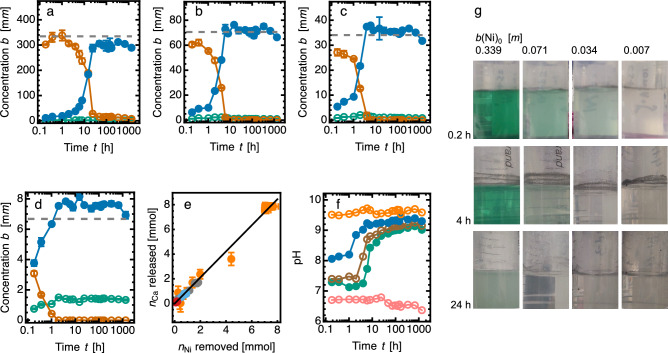
Solution composition during the Ni-CS sequestration reaction. **a**–**d**   Concentrations *b* (m*m*) of Ca (blue), Ni (brown), Si (green), means of three measurements with error bars showing the range; horizontal dotted lines show the initial Ni concentration $b({\textrm{Ni}})_0$(*m*) (**a**) 0.339, (**b**) 0.071, (**c**) 0.034, (**d**) 0.007. (**e**) Amount of Ca released $n_{\textrm{Ca}}$ vs amount of Ni removed $n_{\textrm{Ni}}$: composite plot of data from all solutions; regression line $n_{\textrm{Ca}}= (1.06\pm 0.01) n_{\textrm{Ni}}$. **f** Variation of pH in Ni-CS slurries during sequestration reaction: the initial Ni$\text {(NO}_{3}\text {)}_{2}$ concentration $b({\textrm{Ni}})_0$(*m*) 0.339 (pink), 0.071 (green), (c) 0.034 (brown), (d) 0.007 (blue), 0 control (orange), decreases upwards from the bottom. **g** Photographs of samples (decreasing initial concentration from left to right) taken after centrifuging, loss of colour showing the progressive removal of Ni from solution.

Figures 2a–d show that the removal of Ni *from* solution is accompanied by an equivalent and synchronous release of Ca *into* solution, as found previously for Co^11^. The time for complete removal decreases with decreasing Ni concentration in solution, and takes *≈ * 28 h at the highest concentration used (0.339 *m*). At all times the concentration of dissolved Si is always less than *1.6± 0.1* m*m*  (45 ppm). The initial amount of Ni in the solution of lowest concentration (0.007* m*) is removed in around 1 h (Fig. 2d). The residual Ni after reaction is extremely small. In solutions that remain in contact with excess CS, the mean Ni concentration when the sequestration reaction is complete is below the level for accurate ICP analysis. We take this to show that the residual Ni concentration is at most 0.1 ppm. A valuable baseline is provided by a zero-Ni solution. In this, the Ca concentration is in the range 1.0–1.5 m*m*, while the concentration of dissolved Si is 1.5–3.0 m*m*. These small concentrations (40–80 ppm) are consistent the known solubilities of xonotlite and calcite. The amount of Ni sequestered equals the amount of Ca released in all solutions and at all time steps, as shown in Fig. 2e. Throughout the reaction, the replacement is stoichiometric. We find the ratio of the amounts *n* (mol) of each ion involved in the replacement reaction, $n_{\textrm{Ca}}/n_{\textrm{Ni}} = 1.06\pm 0.01$.

From these results we conclude that sequestration occurs through the formation of a Ni-silicate phase, since the Si concentration in solution does not change during the reaction. We denote this product phase as Ni-S-H, using notation from cement chemistry where S denotes SiO$_{2}$, H denotes H$_{2}$O, and dashes indicate that the stoichiometry is unknown or variable.

#### Variation of pH during sequestration

Figure 2f shows the evolution of pH during the nickel sequestration reaction. In the control sample with no Ni the pH rapidly settles at *≈ 9.6* as a result of the dissolution of xonotlite, a basic and sparingly soluble phase^26–30^. In samples containing Ni the pH is lower at early times. This reflects the fact that Ni$\text {(NO}_{3}\text {)}_{2}$ solutions are weakly acidic as a result of slight hydrolysis of the Ni$^{2+}$ aquo-ion. The initial pH increases with decreasing initial Ni concentration. Ni-S-H formed in the sequestration of Ni has low solubility. In the samples with initial Ni concentration 0.007–0.071 *m* the pH rises sharply when the Ni is completely sequestered, and ultimately stabilises at *≈ *9.0–9.4, the final pH decreasing slightly with increasing Ca$^{2+}$ concentration through the common-ion effect on xonotlite solubility. These samples all contain residual xonotlite. In contrast, the pH of the sample with initial concentration 0.339 *m* remains around 6.5 when the reaction is complete because in this case there is a residual Ni$^{2+}$ concentration of *≈ *0.015 *m* and no xonotlite. The overall evolution of pH during the replacement reaction is similar to that reported previously^11^ for Co, except that the Ni sequestration is a little more rapid.

A recent recalculation of the solubility product of synthetic xonotlite^29^, and confirmed by new measurements^30^, allows some further analysis of the solution compositions during sequestration. The calculated equilibrium pH of xonotlite in water at 25 $^{\circ}$C is 10.57. This value is unaltered in the presence of calcite alone, but is sensitive to trace CO_2_. The observed pH of the control experiment with no Ni is 9.61 (see Fig. 2f), the value calculated for xonotlite/calcite/water system in contact with 0.5 ppm CO$_{2}$(g).

#### Characterisation of the reaction product

**Fig. 3:**
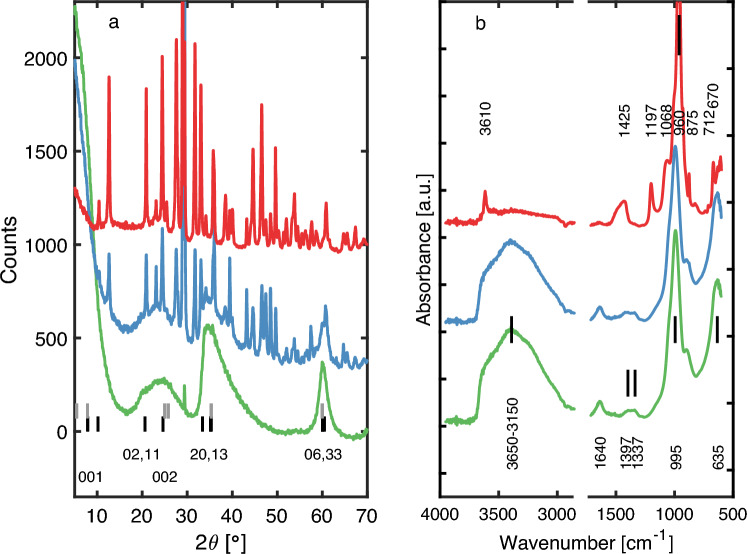
Changes in solid structure during Ni$^{2+}$ sequestration. (**a**) XRD patterns of solid material formed by reaction of CS with Ni$\text {(NO}_{3}\text {)}_{2}$ solution of initial concentration $b({\textrm{Ni}})_0=0.339$ *m* after 2 h (red), 15 h (blue) and 2 d (green); tick marks show reported positions of *hk* and 00*l* Bragg reflections of Ni phyllosilicates and precursors formed at ambient temperatures^31–34^ (black 1:1, grey 2:1 phyllosilicates). (**b**) Mid-IR spectrum of the same solid materials shown in (**a**). Broad weak bands at 1337 and 1397 cm^-1^ are identified as asymmetric stretch modes of adsorbed hydrated NO$_{3}^{-}$ ion^35^. XRD patterns and IR spectra are offset for display.

XRD and FTIR analyses (Fig. 3a, b) show that the product of the CS reaction with solution $b({\textrm{Ni}})_0=0.339$ *m*, in which all Ca initially present is replaced by Ni, is largely amorphous. The reaction is complete after 2 d, after which time the XRD pattern is unchanged over a further 54 d. The only remaining sharp reflection is from minor calcite which does not participate in the sequestration reaction. The xonotlite reflections of the untreated CS are absent in the fully reacted solid. There are three new features not present in xonotlite: a broad strongly asymmetric reflection with a maximum intensity at 34.6$^\circ$ *2θ * (*d*-spacing: 0.259 nm), a narrower less asymmetric reflection centred at 60.2$^\circ$ *2θ * (*d*-spacing: 0.154 nm), and a broad band centred at 24.5° *2θ * (*d*-spacing: 0.361 nm). These features resemble the 2D *hk* diffraction bands described first in disordered layer materials such as graphite^36^, and later in the sheet silicate halloysite^37^. Broad reflections of this kind are found also in synthetic 1:1 and 2:1 Ni phyllosilicates ^31–34,38,39^ that are formed by reacting aqueous solutions of nickel salts with various silicates. The positions and indexing of these reported reflections are shown in Fig. 3a. We index the observed reflections in Ni-S-H correspondingly. The position of the 06,33 band in the Ni-S-H samples matches closely that recorded in several natural Ni-rich 1:1 phyllosilicate minerals, *d*-spacing 0.153 nm and indexed as 060^40^. This similarity suggests that the Ni-S-H reaction product, although highly disordered, contains intact fragments of silicate sheet structures. The XRD pattern of Ni-S-H shows no identifiable basal 00*l* reflections, implying complete turbostratic disorder in the direction normal to the sheets^41^.

We estimate the size $L_a$ of the sheet fragments (strictly the linear dimension of the coherent scattering domain in the *a*-*b* lattice plane) from the width of the 06,33 band^36,37,42–44^ as *≈ 8* nm. We regard this estimate as merely indicative of the sheet size since the exact relation between the domain size and the width of an *hk* band is far from settled^45^. However our estimate of $L_a$ is similar to the mean domain size of 10 nm reported^46^ for C-S-H, the poorly crystalline calcium silicate of Portland cement. From the observed *d*-spacings of the *hk* bands in the reaction product, corrected for sheet size^36,42^, we estimate lattice parameters for the silicate sheet as *a=0.534* nm and *b=0.926* nm (with $b \approx \surd {3}a)$^37,44^. The only available published cell parameters for comparison are of a synthetic 1:1 Ni phyllosilicate heat-treated at 350 $^{\circ}$C with *a=0.534* nm and *b=0.909* nm^47^; and of a low-Mg specimen of the 1:1 Ni phyllosilicate mineral pecoraite with *a=0.527* nm and *b=0.917* nm^48^ (also reported as ICDD 49-1859). There appear to be no available crystal structures of 2:1 pure-Ni phyllosilicates.

Before the time required for full replacement (1.2 d for $b({\textrm{Ni}})_0=0.339$ *m*), Ni-S-H and xonotlite co-exist as shown in Fig. 3a. Likewise, when the solution used does not contain enough Ni to replace all Ca in the xonotlite initially present in 1.00 g of CS (as when $b({\textrm{Ni}})_0=0.007-0.071$ *m*), Ni-S-H and xonotlite co-exist also in the final product.

Figure 3b shows the evolution of the mid-IR spectrum of the solid material as it is converted from CS to Ni-S-H in the Ni/Ca replacement reaction. An important diagnostic is the complete disappearance of the band at 1197 cm$^{-1}$ originally present in the CS spectrum. This band is assigned to the crosslinks in the double-chain structure of xonotlite^49^. Its absence in the fully reacted CS shows that the double-chain structure is dismantled in forming Ni-S-H . The same change occurs in the formation of the Co analogue^11^. The distinct band at 1068 cm$^{-1}$ present in the CS spectrum before reaction (Fig. 1b), and also assigned to a bridging mode^22^, broadens to a shoulder on formation of Ni-S-H . Further evidence of silicate chain disruption comes from changes to the strong band at 960 cm$^{-1}$ in the unreacted xonotlite (Si–O–Si stretching vibrations of non-bridging tetrahedra^22^.) In Ni-S-H this is replaced by a strong, broad band at 995 cm$^{-1}$, assigned to the Si-OH stretching mode in silanol groups^50^. The broad band in the region 3150–3650 cm$^{-1}$ is more prominent in Ni-S-H than in Co-S-H, reflecting its more hydrous character. Likewise the band at 1640 cm$^{-1}$, present only in the reacted product and assigned to the H-O-H bending mode of water^22^, shows that molecular water is present in the product. This feature is also more intense in Ni-S-H than in Co-S-H. The reported mid-IR spectrum of a synthetic 1:1 Ni phyllosilicate (a Ni pecoraite)^33^, shows similar strong Si-O modes at 1005 cm$^{-1}$ and 647 cm$^{-1}$ (995 cm$^{-1}$ and 635 cm$^{-1}$ in Ni-S-H ). There is evidence from the mid-IR spectrum that Ni-S-H is slightly contaminated by nitrate ion^51^.

Taken together, the changes that occur in XRD pattern and mid-IR spectrum show that the xonotlite double-chain structure of CS is destroyed during the sequestration reaction. Both XRD and mid-IR data indicate that the final reaction product has features of a highly disordered Ni phyllosilicate with evidence of fragmentary tetrahedral sheets. The Ni-S-H product formed has end-member loading of Ni (all Ca replaced) and is notably hydrous.

**Fig. 4:**
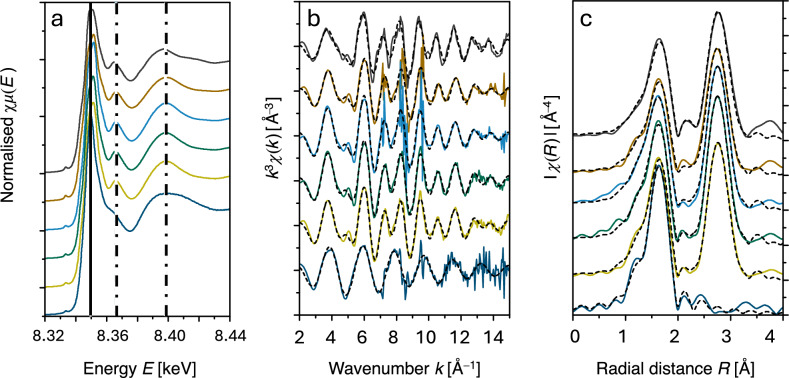
X-ray absorption spectra of Ni standards and Ni-S-H-containing samples formed after 14 d in contact with Ni$\text {(NO}_{3}\text {)}_{2}$ solutions of different initial concentration $b\mathrm {(Ni)_0}$; from the top: $\beta \mathrm{-}\textrm{Ni}\text {(OH)}_2$ standard, $b\mathrm {(Ni)_0}$ = 0.339 *m*, 71  m*m*, 34  m*m*, 7.0  m*m*, Ni$^{2+}$(aq) standard. (**a**) The normalised XANES $\chi \mu (E)$ (solid line marks the Ni ‘white line’); dash-dot lines mark features at *≈ * 8.365 and 8.395 keV. (**b**) the $k{^3}$-weighted EXAFS *χ (k)*, where *k* is the wavenumber; (**c**) the Fourier transform *|χ (R)|* of $k{^3}\chi (k)$.

X-ray absorption spectroscopy [XAS] provides further support for the phyllosilicate character of the Ni-S-H reaction product. Ni K-edge spectra were collected on solid reaction samples and on $$ $$$$$$$$$$$$$$$$$$$$$$$$$$$$$$$$$$$$$$$$$$$$$$$$$$$$$$$$$$$$$$$$ \beta - {\mathrm{Ni(OH)}}_{2} $$ and Ni$$^{2+}$$ (aq) standards to obtain information on the local Ni coordination environment in the reaction product after Ni sequestration. The X-ray absorption near-edge structure (XANES) of the reaction samples (Fig. 4a) has white-line (solid vertical line) and multiple-scattering (dash-dot vertical line) features distinctly different in energy and shape from both the Ni$$^{2+}$$(aq) and the $$\beta -\textrm{Ni}\text {(OH)}_2$$. These variations arise from differences in the local coordination environment of Ni in solid product, and show that the Ni$$^{2+}$$ has lost its primary hydration shell and that the *β *-hydroxide is not present (see Supplementary Information). The XANES of the reaction products from all tested Ni$$^{2+}$$ concentrations are identical, showing the same phyllosilicate structure is formed. The XANES of Ni-S-H closely resembles the Co-edge XANES of Co-S-H previously reported^11^, indicating that the metal ion has a similar local environment in both phyllosilicates.

Further insight into the Ni-S-H structure comes from scattering-path analysis of the EXAFS spectra (Fig. 4b,c). This shows a Ni-O coordination number [CN] of 6 at a radial distance of 2.058-2.063 Å, a Ni-Ni CN 6 at 3.093-3.110 Å, and a Ni-Si CN 4 at 3.231–3.257 Å. These radial distances (Table 1) are similar to those extracted from crystallographic data on the 2:1 Ni-Mg phyllosilicate mineral willemseite^52^, an end-member synthetic 1:1 Ni phyllosilicate^47^ and the previously reported Co-S-H phyllosilicate^11^. This further confirms the reaction product is a Ni phyllosilicate, with no other solid reaction products. The shorter Ni–Ni scattering path and existence of the Ni–Si scattering path in the EXAFS of the solid products show that a *β *-hydroxide is not present. No variations in the coordination numbers the radial distances are identified, confirming again the formation of the same Ni-silicate at all initial Ni$^{2+}$ concentrations.

**Table 1:** Comparison of the radial distance *R* from fits to the Ni EXAFS: extracted radial distances of Ni-S-H compared with those calculated from the crystallographic parameters of the Ni phyllosilicate mineral willemseite^52^, and synthetic Ni phyllosilicates Ni-chrysotile^47^ and Ni-talc^34^; and comparison with the radial distances of Co-S-H formed when Co is sequestered by CS^11^.

	Ni-S-H	Willemseite	Ni-chrysotile^47^	Ni-talc^34^	Co-S-H^11^	
Scattering path	*R* (Å)	*R* (Å)	*R* (Å)	*R* (Å)	Scattering	*R* (Å)
Ni-O	2.058–2.063	2.06–2.08	1.89–2.11	2.055-2.059	Co-O	2.085
Ni-Ni	3.093–3.110	3.05–3.07	2.90–3.21	3.05–3.07	Co-Co	3.13
Ni-Si	3.231–3.257	3.19–3.21	3.21–3.38	3.27–3.29	Co-Si	3.31

#### Mass change on sequestration

**Table 2:** Mass change of CS blocks on complete reaction with nickel nitrate solution at 25 $^{\circ}$C.

Sample	Mass ratio$^a$ $w_a/w_b$	Mol ratio$^b$ $n_{{\textrm H_{2}\textrm O}}/n_\textrm{Xon}$	Mol ratio$^c$ $\Delta n_{{\textrm H_{2}\textrm O}}/n_\textrm{Xon}$	Bulk density$^d$ $\rho _b$ kg/m$^3$	Solid density$^e$ $\rho _s$ kg/m$^3$	Porosity^*f*^ *f*
A	1.507	14.9	4.0	–	–	–
B	1.511	15.0	4.2	–	–	–
C	1.518	15.3	4.1	–	–	–
D	1.510	15.0	4.2	–	–	–
Mean	1.512	15.1	4.1	–	–	–
Replicates (*n=4*)$^g$				425	2345	0.818
CS control				270	2540	0.895

(*a*) Sample weights before reaction ($w_b$), and after reaction ($w_a$). All samples were conditioned over LiCl saturated solution at 25.0 $^{\circ}$C (RH 11.3%^53^) prior to weighing. (*b*) Mol ratio where $n_{{\textrm H_{2}\textrm O}}$ is the water incorporated in the reaction product calculated from weight gain ($w_a-w_b$) after allowing for the mass change associated with replacement of Ca by Ni; and $n_\textrm{Xon}$ is the xonotlite amount before reaction. (*c*) $\Delta n_{{\textrm H_{2}\textrm O}}$ is calculated from weight loss on conditioning the reacted samples over molecular sieve 4A desiccant at 25 $^{\circ}$C (RH < 0.1%)^54^. (*d*–*f* ) Bulk density, solid density and porosity are calculated using the standard Archimedes buoyancy methods^55^ with water as the suspending and saturating liquid; standard uncertainties: $u(\rho _b)$ 4 kg m$^{-3}$, $u(\rho _s)$ 30 kg m$^{-3}$, *u*(*f*) 0.004; quantities $\rho _b$, $\rho _s$, *f* are measured at water activity $a_w=1$, and shrinkage may occur at lower water activity (or lower RH). (*g*) Number *n* of measurements

Our measurements of sample mass indicate that considerable water is taken up during Ni-S-H formation. Tests on small blocks of CS show a mass increase 51% (Table 2) on Ni-treatment. Of this only 16% results from replacing Ca by Ni. Converting CS to Ni-S-H reduces the porosity from 0.90 to 0.82.

From solution data we see that all Ca is replaced by Ni and that no Si is lost from the solid sequestrant, so that the remaining mass gain must come from the incorporation of water, either as molecular water H$_{2}$O or as hydroxyl groups OH or as both. The mass of water in Ni-S-H (here conditioned at 11% RH at 25 $^{\circ}$C) corresponds to *≈ 15* mol water per mol of xonotlite in the initial CS. The reaction stoichiometry is written as:

1 $$ 6{\mathrm{Ni}}^{{2 + }} + {\mathrm{Ca}}_{6} {\mathrm{Si}}_{6} {\mathrm{O}}_{{17}} {\mathrm{(OH)}}_{2} + 15{\mathrm{H}}_{2} {\mathrm{O}} \to {\mathrm{Ni}}_{6} {\mathrm{Si}}_{6} {\mathrm{O}}_{x} {\mathrm{(OH)}}_{{\mathrm{y}}} .{\mathrm{zH}}_{2} {\mathrm{O}} + 6{\mathrm{Ca}}^{{2 + }} {\text{ }} $$

where *x=2+z* and *y=32-2z* by charge balance.

When conditioned over a molecular sieve 4A desiccant samples lose about 20% of the incorporated water. We treat this as a measure of the amount of loosely held molecular water at that RH (<0.1% at 25 $^{\circ}$C), and conclude that most of the incorporated water is chemically combined as OH. Water vapour sorption isotherms show that at 25 $^{\circ}$C Ni-S-H takes up about 10*× * as much water as CS over the RH range 0–80% (see Supplementary Information).

From Table 2, we have *z≈ 4*, so that Ni-S-H has the empirical formula $\text {Ni}_{6}\text {Si}_{6}\text {O}_{6}\text {(OH)}_{24}.4\text {H}_{2}\text {O}$. We write Eq. 1 succinctly as

2 $$\begin{aligned} 6\text {Ni}^{2+} + \text {Xon} +15 \text {H}_2\text {O} \rightarrow \text {Ni-S-H} + 6\text {Ca}^{2+}. \end{aligned}$$

The molecular water combines reversibly, and is non-structural or zeolitic. The hydrous nature of Ni-S-H is evident from the IR bands in the region 3300–3650 cm$^{-1}$ present in Ni-S-H but absent in CS. The weak feature at 1640 cm$^{-1}$ indicates that there is a little molecular water in Ni-S-H at ambient relative humidity, as found gravimetrically. The reaction scheme of Eq. 1 suggests that the double-chain silicate unit (Si$_{6}\mathrm{O}_{17}\text {)}^{-10}$ is destroyed during the reaction. This is confirmed by the disappearance of the IR band at 1197 cm$^{-1}$ which was earlier assigned to crosslinks between the xonotlite double chains. Taken overall, our observations show that the structure is hydrated (IR, mass change), poorly ordered (XRD, IR, XAS), and resembles phyllosilicate fragments (XRD, XAS). These fragments should be edge-terminated with OH groups for charge neutrality. We know the elemental composition of Ni-S-H from that of the xonotlite sequestrant, the stoichiometry of the Ca/Ni exchange reaction, and the mass changes during Ni-S-H formation. Combined with charge-balance constraints this leads to Eq. 1. To find *z* we use the dehydration mass change (*z≈ 4*), which from Eq. 2 gives the empirical formula. This formula for Ni-S-H resembles that reported previously^11^ for the Co compound Co-S-H, Co$_{6}\text {Si}_{6}\text {O}_{7}\text {(OH)}_{22}.3\text {H}_{2}\text {O}$, except that Ni-S-H is slightly more hydrous. We note that accurate structural information has not yet been determined for Co-S-H or Ni-S-H.

The physical properties of Ni-S-H given in Table 2 are similar to those we reported^11^ for Co-S-H. However the solid density of Ni-S-H (2345 kg m$^{-3}$) is about 13% lower than that of Co-S-H (2690 kg m$^{-3}$). We attribute this difference to the somewhat greater amount of water incorporated in Ni-S-H (15 mol H$_{2}$O per mol Xon) than in Co-S-H (13 mol H$_{2}$O per mol Xon). Likewise the porosity of Ni-S-H produced in the CS replacement reaction (*f *0.82) is a little lower than that estimated for Co-S-H (0.85). The amount of water taken up in forming Ni-S-H is greater than in forming Co-S-H, so we expect somewhat greater expansion, and consequently lower porosity.

**Fig. 5:**
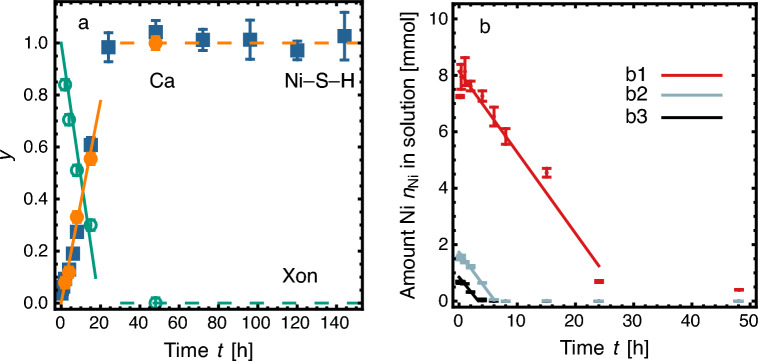
Kinetics of Ni sequestration by CS in stirred powder reactions (25 mL solution mixed with 1.00 g CS powder) at 25 $^{\circ}$C. (**a**) Initial Ni concentration ($b({\textrm{Ni}})_0$ =0.339 *m*): release of Ca$^{2+}$ into solution (Ca, blue points, ICP measurement), decrease of crystalline xonotlite (Xon, green points, XRD measurement), and growth of amorphous Ni-S-H phase (Ni-S-H, orange points, XRD measurement); *y* denotes fractional change. (**b**) Decrease of Ni$^{2+}$ in solution at three initial molal concentrations: b$_1$ 0.339 *m* (red points), b$_2$ 0.071 *m* (grey points), b$_3$ 0.034 *m* (black points); dashed lines are linear fits to data, with best-fit rate constant *k = 0.289± 0.022*, *0.279± 0.009* and *0.254± 0.055* mol/(kg CS h) respectively for the three concentrations; mean *0.275± 0.020* mol/(kg CS h).

#### Kinetics of sequestration

We determine the sequestration kinetics from the ICP and quantitative XRD data. In Fig. 5a we show that in contact with Ni solution a given quantity of CS releases Ca into solution at a constant rate. The increase in the amount of Ca in solution tracks closely the disappearance of crystalline xonotlite in the CS as measured by quantitative XRD (Fig. 5a). It also tracks the appearance of the largely amorphous Ni-S-H phase. The constant reaction rate continues down to low Ni concentrations, until the reaction ceases abruptly, either because all the xonotlite has been consumed or because all the Ni has been sequestered.

In Fig. 5a, where the initial Ni concentration is 0.339 m, the timescale for complete release of Ca and complete decomposition of xonotlite is about 28 h. CS sequesters Ni about four times more rapidly than Co. The total amount of Ca released into solution is *7.55± 0.45*  mmol. The initial amount of xonotlite present in the solid CS is 1.30 mmol. This mol ratio (7.55/1.30) = *5.8± 0.3* is close to the expected value of 6 for stoichiometric Ni/Ca replacement.

How the amount of Ni in solution decreases with time is a direct measure of Ni sequestration rate. In Fig. 5b we show this relation for three different initial Ni concentrations. As noted previously for Co^11^ (and equally remarkably) the rate is the same for all concentrations. This means that the rate of sequestration is independent of the initial Ni concentration over a tenfold variation, and that it remains independent of Ni concentration to the lowest levels we have measured. Therefore the reaction has zero-order kinetics with respect to Ni. It is likely that the Ni sequestration rate is determined by the decomposition or dissolution of xonotlite through the rate at which Ca is released into solution. For Ni sequestration we have

3 $$\begin{aligned} {\mathrm d}n_\textrm{Ni}/{\mathrm d}t= -m_\textrm{CS} k, \end{aligned}$$

or

4 $$\begin{aligned} n_\textrm{Ni}(t)=n_\textrm{Ni}(0)-m_\textrm{CS}kt, \end{aligned}$$

where $n_\textrm{Ni}$ is amount of Ni (mol) in solution, $m_\textrm{CS}$ the mass of CS sequestrant, and *k* the rate constant. Here the best-fit rate constant $k_0=0.275\pm 0.020$  mol/(kg CS h) at 25 $^{\circ}$C. This translates to a practical rate of removal in a well-stirred batch reactor of 385 kg Ni per tonne of CS per day. The quantity $m_\textrm{CS}$ is likely to be a proxy for the Si content of the solid CS, which is conserved and constant throughout the sequestration process. The amount of Ni-S-H produced is therefore proportional to the amount of Si present in quantity of CS used.

The time for the complete removal of Ni from solution is proportional to the initial amount of Ni in solution, irrespective of concentration. This is characteristic of a zero-order reaction. The clearance times are *27.5± 2,**6.2± 0.3* and *3.3± 0.8* h for equal volumes of solution at the three initial Ni concentrations $b({\textrm{Ni}})_0$ 0.339, 0.071 and 0.034 *m*, each in contact with 1.00 g CS at 25 $^{\circ}$C. Generally, in these tests the time for Ni removal is $81.1\,(b({\textrm{Ni}})_0/m)$ h. The rate constant no doubt depends on the particle size of the CS sequestrant, as well as on temperature.

### Reaction-diffusion in CS packed beds

Recently, we described^11^ a novel experimental arrangement in which we observed the advance of a reaction zone in a packed bed of CS brought into contact with a solution containing Co^2+^ ion. The relatively rapid sequestration reaction and the slower diffusion combine to produce a reaction zone with a well-defined sharp front. Experimental data were described accurately by the Sharp Front [SF] reactive transport model we set out.

**Fig. 6:**
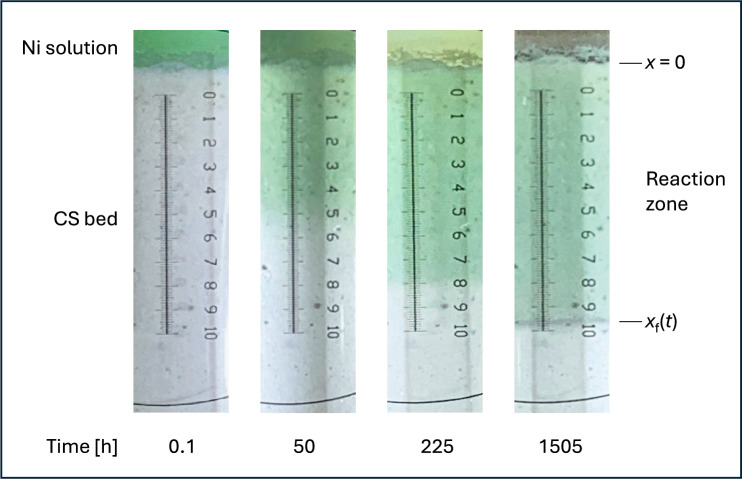
Sharp Front experiment: Time-series photographs of penetration of Ni$^{2+}$ into a CS bed at 25 $^{\circ}$C, showing the well-defined reaction zone (green) and reaction front at $x=x_\textrm{f}$. The CS bed is contained in a flat-bottomed glass tube, i.d. 10.44 mm, 10 mm graticule attached (full width of tube not shown). The initial Ni$\text {(NO}_{3}\text {)}_{2}$ concentration = 0.33* m* and CS bed volume fraction porosity $f_\textrm{b}$ = 0.93. The CS bed was pre-saturated with water to suppress capillary transport. The image at the right taken at *t=* 1505 h shows the reaction front position $x_{\mathrm f\infty }$ when the sequestration reaction is complete.

We have now carried out similar tests on Ni$^{2+}$ using the same packed-bed arrangement. The experimental arrangement, described fully in^11^, is shown in Fig. 6. The SF model provided an excellent description of SF experiments with Co$\text {(NO}_{3}\text {)}_2$ solutions, and we find that the model represents the results with Ni$\text {(NO}_{3}\text {)}_{2}$ solutions equally well.

As shown previously^11^, the position $x_\mathrm f$ of the reaction front at time *t* is given by

5 $$\begin{aligned} -x_{\mathrm f} - x_{\mathrm f\infty } \ln \bigl [1-\frac{x_{\mathrm f}}{x_{\mathrm f\infty }}\bigr ] = \frac{K}{L_{\mathrm s}} t. \end{aligned}$$

Here $x_{\mathrm f\infty }$ is the final position of the reaction front when the sequestration is complete, $L_{\mathrm s}$ is the length occupied by the solution of concentration *c* in the tube of cross-section area *A* and *K* is a transport parameter, dimension ${\mathsf L^{2}} {\mathsf T^{-1}}$ as a diffusivity.

The non-dimensional form of Eq. 5 with $X=x_{\mathrm f}/x_{\mathrm f\infty }$, and $T= K t/(x_{\mathrm f\infty } L_{\mathrm s})$ is

6 $$\begin{aligned} -X - \ln (1-X)=T, \end{aligned}$$

with $X=(2T)^{1/2}$ at early times.

**Fig. 7:**
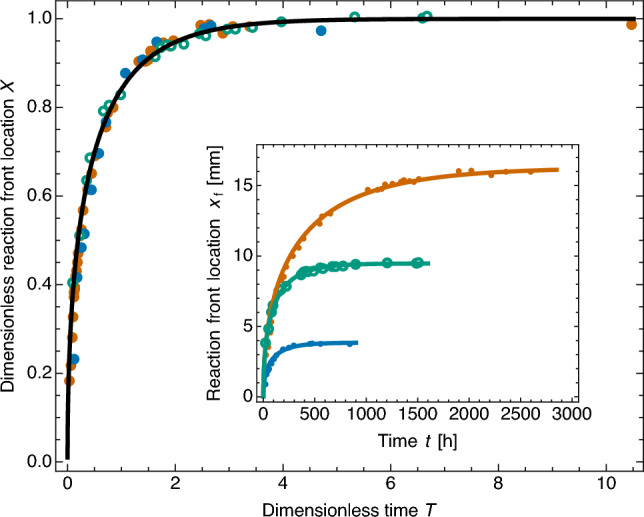
Advance of the Ni sequestration front in CS packed beds: three experimental datasets scaled to the dimensionless form of the SF model, Eq. 6 (−). Orange filled circles, $b({\textrm{Ni}})_0 = 0.332\,$*m* ($c({\textrm{Ni}})_0 = 0.328$ mol/L), $L_{\mathrm s}=67.7$ mm, $\rho _\textrm{b}=200$ kg/m$^3$; green open circles, $b({\textrm{Ni}})_0 = 0.332\,$*m* ($c({\textrm{Ni}})_0 = 0.328$ mol/L), $L_{\mathrm s}=35.0$ mm, $\rho _\textrm{b}=185$ kg/m$^3$; blue filled circles, $b({\textrm{Ni}})_0 = 0.158\,$m ($c({\textrm{Ni}})_0 = 0.157$ mol/L), $L_{\mathrm s}=38.5$ mm, $\rho _\textrm{b}=240$ kg/m$^3$. Inset shows the unscaled experimental data ($x_{\mathrm f},t$) fitted to Eq. 5. Fit parameters: orange filled circles, $\alpha _0=47.23\pm 3.42$ mm$^{-1}$ h, $\alpha _1=16.27\pm 0.12$ mm; green open circles, $\alpha _0=23.83\pm 1.40$ mm$^{-1}$ h, $\alpha _1=9.47\pm 0.05$ mm; blue circles, $\alpha _0=46.35\pm 10.01$ mm$^{-1}$ h, $\alpha _1=3.85\pm 0.10$ mm.

In experimental tests, the position $x_{\mathrm f}$ of the reaction front was measured from photographic images as described in Methods below. Figure 7 (inset) shows experimental data on the advance of the Ni$^{2+}$ sequestration front in three SF tests in which the molality of the Ni$\text {(NO}_{3}\text {)}_{2}$ solution was varied. There was also a small variation in packing density of the CS bed, which was formed by sedimentation from a dispersion of CS in water, followed by removal of syneresis water by conditioning in a saturated LiCl humidistat. Tests were allowed to run for times much greater than needed to achieve complete sequestration to observe the long-term stability of the reaction zone. In Fig. 7 (inset) the data are fitted to the SF model, Eq. 5. The scaled data (*X*, *T*) collapse on to a single curve, which is described well by the dimensionless form of the SF model, Eq. 6. This provides the master curve for the sequestration kinetics, as was also the case for Co^11^.

The success of the SF model in representing experimental data confirms the main assumption of the model: namely, that Ni sequestration in the SF tests is controlled by the slow Ni$^{2+}$ ion diffusion in the bed, not by the much faster kinetics of the sequestration reaction that was measured in the stirred-batch experiments using CS powder.

As shown previously^11^, the SF model has only two disposable fit parameters, $\alpha _0$ and $\alpha _1$. In fact, $\alpha _1= x_{\mathrm f \infty }$, the final location of the reaction front, and this is obtained by direct measurement with little error at the end of the experimental run. Since $L_{\mathrm s}$ is known, *K* can be determined immediately from the fit parameter $\alpha _0=L_{\mathrm s}/K$. From the data of this test is found to be $(4.30\pm 0.25)\times 10^{-10}$ m$^2$ s$^{-1}$.

**Fig. 8:**
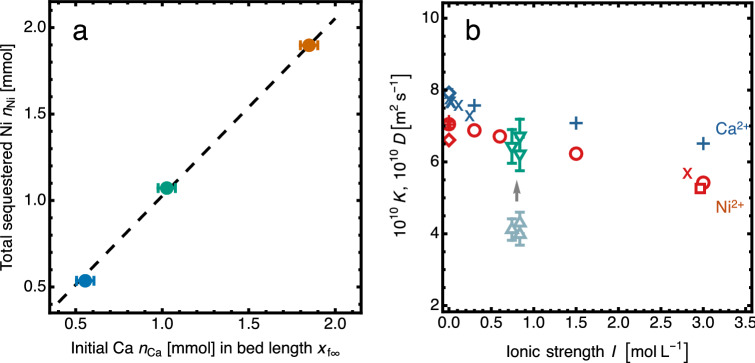
Analysis of three experimental tests of Ni sequestration by replacement of Ca in CS beds, with different bed lengths and Ni amounts. (**a**): Replacement of Ca by Ni at equilibrium, dashed line $n_\textrm{Ni} = (1.03 \pm 0.01)n_\textrm{Ca}$ (marker colours as Fig. 7; uncertainties shown graphically). (**b**) Sharp Front transport parameter *K* estimated from experimental data (up triangles) plotted vs ionic strength *I*; porosity-corrected $K^\prime$ (down triangles); published diffusivity data for Ni$^{2+}$ and Ca$^{2+}$ ions in aqueous solution (25 $^{\circ}$C, interpolated where necessary) Ni$^{2+}$ (red symbols)^56–59^, Ca$^{2+}$ (blue symbols)^59–61^.

The Ni:Ca stoichiometry of the reaction is derived directly from the fit parameter $\alpha _1$. Since $\alpha _1 = x_{\mathrm f \infty }$, then $\gamma =\alpha _1/m_{\mathrm s0}$, the ratio of the final length of the reaction zone to the initial mass of Ni in solution. The packing density of the bed $\rho _\textrm{b}$ is known, and $1/(A\gamma \rho _\textrm{b})$ is the mass of Ni sequestered per unit mass of CS. We then compare the total amount (mol) of Ni sequestered with the amount of xonotlite Ca originally present in the length $\alpha _1=x_{\mathrm f\infty },$ the equilibrium location of the reaction front. Figure 8a shows that this mol ratio is 1.03*± 0.01.* All the Ca available in the CS in the reaction zone is replaced by Ni, and the Ca/Ni replacement is stoichiometric and complete. This confirms the same result from solutions analysis of stirred systems using powdered CS.

#### The SF transport parameter and the diffusivity

Previously^11^ we identified the SF transport parameter *K* with the diffusivity of the Co$^{2+}$ ion in the reaction zone. We now describe the transport processes in the SF experiment more fully. The primary process of interest is the diffusion of Ni$^{2+}$ from the bed surface to the SF, the front of the reaction zone at location $x_\mathrm f$. Since the Ni-Ca replacement reaction rate is rapid compared with the diffusion rate, the front acts as a sink for Ni$^{2+}$. Correspondingly, the front acts as a source of equal strength for Ca$^{2+}$, which on release diffuses back towards the bed surface. Electroneutrality requires that the diffusion rates of the two cations are the same, and that there is no net diffusion of the NO$_{3}^{-}$ anion. However, in addition to this main process, there is also some diffusion of Ca$\text {(NO}_{3}\text {)}_{2}$ from the reaction zone into the unreacted CS bed beyond the front. The effect of this is to reduce the ionic strength of the supernatant solution from its initial value $I_0$ to a final value $I_\infty$ when all Ni$^{2+}$ ions have been sequestered and the reaction is complete. In our SF tests, $I_0$ is typically 0.7–0.9 mol L$^{-1}$ and $I_\infty$ 0.6–0.7 mol L$^{-1}$. Thus if we identify the parameter *K* as the Ni$^{2+}$ ion diffusivity, we are measuring it in a porous medium at relatively high ionic strength, and in the unusual circumstance of coupled counter-diffusion with Ca$^{2+}$.

The reaction zone through which the cations diffuse consists of fully converted Ni-S-H . We estimate the porosity of the zone $f_{\mathrm z}$ by using the result of Table 1, which shows that the porosity *f* of CS is reduced by about 10% on conversion to Ni-S-H . The initial porosity of the packed bed is known. A simple $1/f_{\mathrm z}^2$ extrapolation^62^ of *K* to $f_{\mathrm z}=1$ then removes the resistance factor arising from the porosity and tortuosity of the bed, and provides an estimate of the Ni$^{2+}$ ion diffusivity in aqueous solution at the same ionic strength. We find from three SF tests that $K^\prime =K/f_{\mathrm z}^2 \approx 6.5 \pm 0.3 \times 10^{-10}$ m$^2$ s$^{-1}$. The *K* and $K^\prime$ values from SF tests are plotted in Fig. 8b, where we also show some published data on Ni$^{2+}$ and Ca$^{2+}$ ion diffusion in aqueous salt solutions. Data are sparse, but diffusivities have been reported for aqueous NiCl$_{2}$ and Ni$\text {(ClO}_{4}\text {)}_{2}$ solutions of several concentrations, some in ternary systems^56–58,63^. Figure 8b also shows available data on the Ca$^{2+}$ ion diffusivity in aqueous CaCl$_{2}$, which is only a little higher than that of Ni$^{2+}$, so that the effect of coupled counter-diffusion is likely to be small. Our estimated value of $K^\prime$ lies approximately between the values of the ion diffusivities of Ni$^{2+}$ and Ca$^{2+}$ at the ionic strength used in the SF tests.

### Comparing the Ni/CS and Co/CS reactions and reaction products

**Table 3:** Comparison of XRD parameters of Ni-S-H and Co-S-H reaction products (Ni-S-H data, this paper; Co-S-H data from^11^).

	Ni-S-H		Co-S-H		
*hk* band	02,13	06,33	20,13	06,33	
*2θ * uncorrected [$^\circ$]	34.63	60.16	34.35	59.40	
*d*-spacing uncorrected [nm]	0.259	0.154	0.261	0.156	
Sheet size $L_a$ [nm]	2.9	8.8	2.7	8.1	Note *a*
*2θ * corrected [$^\circ$]	33.67	59.34	33.31	59.05	Note *b*
*d*-spacing corrected [nm]	0.266	0.154	0.269	0.156	
Width FWHM [$^\circ$ *2θ ] *	2.9	8.8	2.7	8.1	
Asymmetry *As*	3.1	1.3	3.3	1.1	Note *c*
Cell parameters *a*, *b* [nm] from uncorrected *d*-spacings	0.529, 0.921		0.535, 0.932		Note *d*
Cell parameters *a*, *b* [nm] from corrected *d*-spacings	0.534, 0.926		0.541, 0.938		

**Fig. 9:**
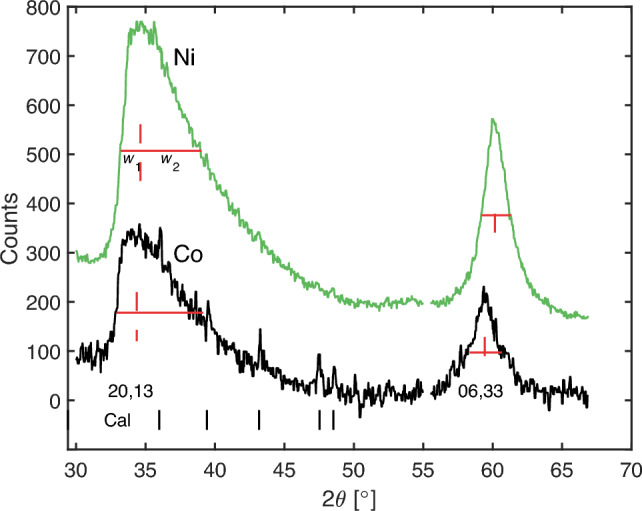
The 20,13 and 06,33 bands of Ni-S-H and Co-S-H compared. Ni-S-H data as Fig. 1; Co-S-H data from^11^. The quantities $w_1$ and $w_2$ mark the low-angle and high-angle half-widths of the peak at half-maximum intensity. The dotted vertical lines indicate the best-estimate positions of the band maxima. Prominent peaks of minor component calcite Cal are shown. The Ni pattern is displaced upwards by 200 counts for display.

(a) Calculated from Warren formula^36,44^

(b) Corrected for finite size $L_a$ of sheet^37,42^

(c) *As* calculated^64^ as ratio of high-angle half-width/low-angle half-width $w_2/w_1$ (see Fig. 9)

(d) Assumes orthogonal setting of hexagonal lattice^44^

Our results show that the sequestration of aqueous Ni$^{2+}$ by CS resembles that of Co$^{2+}$ in most respects. The notable differences are (1) that (subject to some uncertainty in CS particle-size control) the Ni$^{2+}$ reaction proceeds about 3.7 times faster than the corresponding Co$^{2+}$ reaction at all concentrations; (2) that the reaction product Ni-S-H is somewhat more hydrous than Co-S-H; and (3) that the specific surface area of Ni-S-H is about 30% greater than that of Co-S-H prepared from the same CS starting material (see Supplementary Information, section S6). The mid-IR spectra of Ni-S-H and Co-S-H are similar, but with a stronger band in the water region in Ni-S-H and a small shift in the silanol band to 995 cm$^{-1}$ from 985 cm$^{-1}$. XRD data of Ni-S-H and Co-S-H show similar phyllosilicate features, with only small differences. Table 3 compares measured, estimated and calculated quantities. The *hk* bands in the XRD patterns of Ni-S-H and Co-S-H are shown together in Fig. 9. The XAS data further confirm the similarity of Ni-S-H and Co-S-H. The radial distances in Ni-S-H are slightly shorter than those in Co-S-H (see Supplementary Information Table S4). We attribute this to the small difference in the crystal ionic radii of Ni and Co in octahedral coordination (Ni$^{2+}$ 0.830 Å, Co$^{2+}$ 0.885 Å)^65,66^. The difference in radial distances from XAS is consistent with the slightly smaller cell parameters for Ni-S-H from the XRD data (Table 3).

**Fig. 10:**
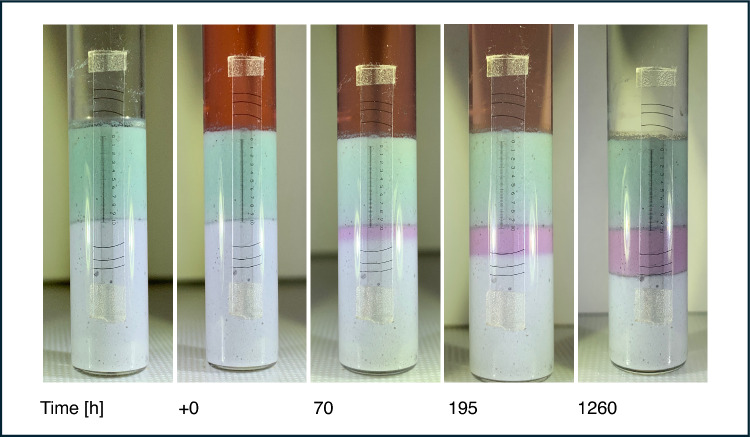
Time-series images showing the diffusion of Co$^{2+}$ through a preformed bed of Ni-S-H. From left to right: the preformed Ni-S-H reaction zone; a solution of Co$\text {(NO}_{3}\text {)}_{2}$ solution ($b({\textrm{Co}})_0 = 0.331\,$*m*) added above the bed; and three images at different later times showing the development of a Co-S-H reaction zone beyond the Ni-S-H zone.

### Ni, Co sequential sequestration in a CS packed bed

The SF experimental design provides a simple but highly visual means to observe additional features of the CS sequestration reaction. For example, Fig. 10 shows the progress of a sequential reaction in which first Ni$^{2+}$ and then Co$^{2+}$ diffuse into a CS packed bed. We find that the Co$^{2+}$ ions diffuse through the Ni-S-H reaction zone without displacing the Ni, but are themselves sequestered when they reach the unreacted CS beyond the Ni-S-H . A sequential test in which the Co precedes the Ni shows similar results: the Ni passes through the Co-S-H zone without displacing the Co but is captured further down the bed by unreacted CS. In the case of Fig. 10, the ratio of the lengths of the Ni-S-H and Co-S-H reaction zone after complete sequestration is 1.57, the same within experimental uncertainty as the ratio of the amounts of Ni and Co used in the test, 1.55.

### Conclusions

- We demonstrate that the commercial calcium silicate hydrate material CS has a sequestration capacity for aqueous Ni$^{2+}$ similar to that we have shown previously for aqueous Co$^{2+}$^11^. This is not unexpected given the general chemical similarity of transition metals Co and Ni, although we note that the CS sequestration rate of Ni is about 3.7 times faster than that of Co at all concentrations. As with Co, Ni is sequestered down to low ppm residual levels and commonly occurring ions do not interfere with the reaction. We also show that Ni is not displaced by Co from Ni-S-H once formed.
- The sequestration of Ni occurs spontaneously at ambient temperature, and removes 0.44 kg of Ni per kg of CS at ambient temperature in less than 2 days under well mixed conditions. Other materials that can remove Ni from aqueous solution are at least *× *10 less effective (and usually much less) and act by surface or interlayer sorption^67,68^. The reaction product, here denoted Ni-S-H , is a poorly crystalline hydrous Ni phyllosilicate of high surface area, in which Ni$^{2+}$ has replaced Ca$^{2+}$ in the CS sequestrant.
- These results show that the various applications that we identified for CS sequestration of Co$^{2+}$ can be extended to Ni$^{2+}$. Of particular potential are the use of CS in filtration or reactor processes for water clean-up, mine waste recovery and recycling. The use of the Ni-S-H reaction product as a direct precursor for water-splitting^8^ or reforming catalysts^69^ may have even greater value for Ni than for Co.
- Results from new packed bed tests confirm the applicability of the Sharp Front reactive transport model, and show that an ion diffusivity can be estimated.

## Methods

CS materials, preparation and analysis of solutions, methods of characterising solids and treatment of errors were generally similar to those used in our companion paper on cobalt sequestration^11^. A journal rule prevents the authors from describing methods in the same words as previously^11^, but the reader should understand that a difference in wording does not imply a difference in method.

### Materials

The CS sequestrant was the commercial product Calsitherm insulation board (Calsitherm Silikatbaustoffe GmbH, Paderborn), manufactured by steam autoclaving lime and silica at 150–200 $^{\circ}$C for 15–25 h^70,71^.

### Preparation and analysis of solutions

Procedures were generally as described previously^11^. Solutions for ICP analysis were prepared by volume. We report solution composition here as molality *b* (unit mol/kg water, denoted mol/kgw or* m*). In transport models we use the amount concentration *c* (mol/m$^3$) and the mass concentration $c^\prime =c M_\textrm{A}$ (kg A/m$^3$), where $M_\textrm{A}$ is the molar mass of substance A, also as in^11^. In batch experiments 1.00 g of CS was combined in 50 mL tubes with 25.0 mL of Ni(NO_3_)_2_ solution of concentration from 0.007 *m* to 0.339 *m*. The samples were then mixed on a shaker-tray for various times ranging from 10 min to 56 d. The final slurry pH was recorded. After centrifugation, the supernatant was extracted, acidified, and diluted using 3% HNO$_{3}$, and then split into triplicates for ICP-OES analysis. The residue was washed, centrifuged again, and then dried at 40 $^{\circ}$C to constant weight.

### Characterisation of solids

#### Total carbon [TC] and total organic carbon [TOC] analyses

Elemental Microanalysis Ltd (Devon, UK) carried out the TC and TOC analyses. For TC analysis, samples dried at 40 $^{\circ}$C were weighed into silver capsules and burned in oxygen at 1000 $^{\circ}$C. The product gases were passed over catalysts for complete oxidation and to eliminate halogen and sulphur interferences. CO$_{2}$ was quantified chromatographically. For TOC analysis samples were treated with 15 m HCl to eliminate any carbonates present.

#### X-ray fluorescence [XRF]

Materials were analysed on a PANalytical AXIOS wavelength-dispersive spectrometer by AMG Analytical Services Ltd. Powder samples were prepared by fusion into glass beads with $\text {Li}_{2}\text {B}_{4}\text {O}_{7}$ at 1270 $^{\circ}$C. Six certified reference materials were used for calibration.

#### X-ray diffraction [XRD]

All XRD procedures were consistent with those used previously^11^. Powder patterns were acquired using a PANalytical Empyrean instrument operating in reflection mode (Bragg-BrentanoHD module) and equipped with a Cu anode working at 40 mA and 45 kV. Instrument settings (slits, Soller) were optimised to prevent beam spillover from the 16 mm dia sample. The incident beam optics comprised primary (14 mm) and secondary (6 mm) masks, a 0.125$^\circ$ fixed-divergence slit, and a 0.03 rad Soller (iCore). The divergent-beam side employed a 0.125$^\circ$ anti-scatter slit and a 0.04 rad Soller (dCore). Back-loading holders were used on powdered samples, over a 2*θ * range of 5–80$^\circ$ (step size 0.0263$^\circ$ 2*θ *, count time 2 s/step) while spinning at 2 rev/s. TOPAS v5 and EVA v6.0 (Bruker Ltd) were used for identification and quantification of phases. Phase quantification relied on the PONKCS method^72,73^. An ‘hkl phase’  (with PONKCS-style cell mass) was developed using calibration mixtures containing a ZnO standard (Sigma-Aldrich, >99.9%).

#### Fourier transform infrared spectroscopy [FTIR]

Mid-IR spectra were obtained with an Agilent 4500a instrument. For each powdered sample, 64 scans were accumulated at a resolution of 4 cm$^{-1}$.

#### X-ray absorption spectroscopy [XAS]

All procedures were consistent with those used previously^11^. XAS was performed at beamline B18 (Diamond Light Source) on solids obtained from batch reactions at all initial Ni concentrations after 56 d of reaction, as well as on $\beta \textrm{-Ni}\text {(OH)}_2$ and Ni$^{2+}$(aq) standards. Dried solids were diluted with BN and pressed into pellets (0.1–0.4 mm thickness). Ni K-edge (8.333 keV) data were collected using a Si(111) monochromator. Transmission mode was used for the $\beta \textrm{-Ni}\text {(OH)}_2$ standard and the higher concentration samples ($b({\textrm{Ni}})_0 =$ 0.339 *m* and 0.071 *m*). Fluorescence mode, employing a 36-element Ge detector, was used for the aqueous Ni${}^{2+}$ standard and the lower-concentration samples ($b({\textrm{Ni}})_0 =$ 0.035 *m* and 0.007 *m*). We used Demeter software for spectral processing and Athena for data reduction, with Artemis for fitting the *R*-space Fourier transform of the EXAFS^74^.

### Reaction front analysis

The positions of the reaction front $x_\textrm{f}$ were extracted from photo images of the CS bed. There was no change in the position of the bed surface during the reaction, within measurement precision, $\approx \pm 0.02$ mm. To measure the front location $x_{\mathrm f}$ each image was calibrated individually from the 10 mm graticule fixed to the tube. For fitting $x_\textrm{f}(t)$ data, Eq. 5 may be written^11^ as

7 $$\begin{aligned} t= \alpha _0[-x_\textrm{f}-\alpha _1\ln (1-x_\textrm{f}/\alpha _1)] \end{aligned}$$

with regression parameters $\alpha _0=L_\textrm{s}/K$ and $\alpha _1=x_{\mathrm f \infty }$.

### Uncertainty analysis

All procedures were consistent with those used previously^11^. Errors in measured quantities are either type A *k=2* expanded uncertainties estimated from standard deviations of replicates or type B estimates based on the known accuracy of laboratory measurements. Errors in least-squares regression parameters are *k=2* uncertainties calculated from standard errors of regression with 0.95 confidence interval. Errors in derived quantities are estimated by standard error propagation methods.

## Supplementary Information

Below is the link to the electronic supplementary material.

Supplementary Material 1

## Data Availability

The datasets generated during and/or analysed during the current study are available from the corresponding author on reasonable request.
