# Supplementary material for: A low-cost calcium silicate hydrate with an exceptionally large sequestration capacity for aqueous divalent nickel
Source: Sci Rep. 2026 Jul 7;16:20997. doi: 10.1038/s41598-026-51622-8 (PMC13342668; doi:10.1038/s41598-026-51622-8)
Supplement: Supplementary file 1 — Supplementary Material 1 [file 41598_2026_51622_MOESM1_ESM.pdf]

A low-cost calcium silicate hydrate with an exceptionally large sequestration capacity for aqueous divalent nickel

Han Zhou, Pieter Bots, Bao Liu, Christopher Hall, Andrea Hamilton

Supplementary information

S1 Experimental notes

Materials

As in[1], the Calsitherm CS used was taken from two stocks of materials: CS-1, material distributed to participants in the Hamstad round-robin project[2] (bulk density ρ_b 270 kg m^{-3}, solid density ρ_s 2540 kg m^{-3}); and CS-2, newly obtained commercial material (ρ_b 185 kg m^{-3}). The mineral composition of the two CS materials was found by XRF and XRD analyses to be similar: CS-1, 94 wt percent xonotlite, 6 wt percent calcite; and CS-2 89 wt percent xonotlite, 11 wt percent calcite. Both materials contain small amounts of cellulose fibres, approximately CS-1 (2.4 wt percent cellulose) and CS-2 (3.6 wt percent cellulose) by TC and TOC analyses. The physical and mineralogical properties of CS have been published previously.[2–7] In stirred-batch and SF tests the chemical behaviour of CS-1 and CS-2 was indistinguishable.

Powdered CS-1 and CS-2 material was prepared for stirred-batch and SF experiments and for XRD, FTIR and other methods of characterisation in different laboratories using different methods. Some variation in particle size no doubt occurs.

SF reaction front analysis[1]

To ensure that x_f is the dependent variable in obtaining least-squares estimates of α_0 and α_1 we use as nonlinear fit model the inverse of Equation 7 of the main paper

$$x_\mathrm{f} = \alpha_0\big[1 + W[-\exp(-1 - t/(\alpha_0\alpha_1))]\big], \tag{S1}$$

where W is the principal branch of the Lambert W function.

S2 Characterisation of CS material

Oxide analysis by XRF

Table S1 X-ray fluorescence analysis of CS-2. Oxide is reported as wt percent. Loss on ignition, carried out on a separate CS sample, is reported for 450 °C and 1000 °C.

Oxide or loss on ignition (LOI)	Wt percent
Na_2O	< 0.05
MgO	0.51
Al_2O_3	0.16
SiO_2	45.69
P_2O_5	< 0.05
K_2SO_4	0.05
CaO	45.60
TiO_2	<0.05
Mn_3O_4	<0.05
V_2O_5	<0.05
Cr_2O_3	<0.05
Fe_2O_3	0.10
BaO	<0.05
ZrO_2	<0.05
ZnO	<0.05
SrO	<0.05
LOI (450 °C)	2.86
LOI (1000 °C)	7.41

Total carbon analysis

Three sub-samples were analysed (Table S2). The average result is reported in the main paper. Total carbon was analysed by sample combustion followed by carbon gas separation on a chromatography column with a thermal conductivity detector. For organic carbon analysis, separate samples were acidified by hydrochloric acid digestion at 80°C to remove inorganic carbonates. The only inorganic carbonate present in CS-2 by XRD analysis is calcite. Calcite quantity (6.33 wt percent) is calculated from TC-TOC and cellulose (2.40 wt percent) calculated from TOC.

Table S2 Carbon analysis of CS-2.

Carbon analysis	wt percent		
Total carbon	1.87	1.81	1.80
Total organic carbon	1.06	1.07	1.07

SEM views of CS microstructure

S3 Gravimetric sequestration data

Table S3 reports raw weight data on four CS blocks that have reacted completely with excess aqueous $Ni(NO_3)_2$ solution (0.33 mol/kgw) at 25 °C. These data are used to estimate the empirical formula of Ni-S-H, as discussed in subsection ***Mass change on sequestration*** of the main paper.

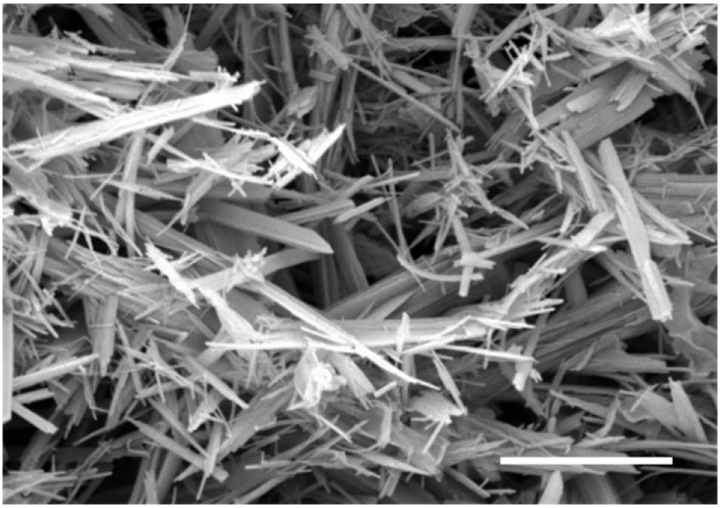

Figure S1 Scanning electron microscope image of CS-1 material captured using secondary electron detector on carbon-coated unpolished sample. Scale bar 2 μm.

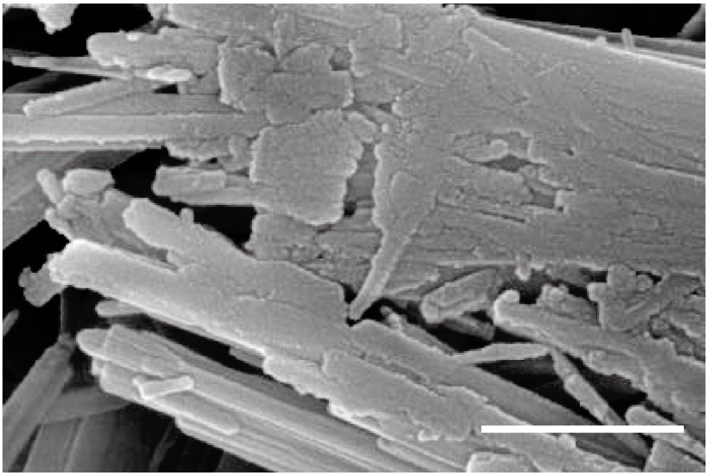

Figure S2 SEM image of CS-1 material showing detail of an individual xonotlite fibre after the material was cut using a scalpel. Scale bar 500 nm.

S4 X-ray absorption spectroscopy [XAS] of Ni-S-H

A shell-by-shell method was used in fitting the spectra to obtain information on the local Ni coordination environment. FEFF6[10] was used to calculate the theoretical (initial) scattering paths from crystallographic data on the

Table S3 Mass change of CS blocks on complete reaction with Ni^{2+}

Sample	Sample wt (g) before reaction	Sample wt (g) after reaction	Sample wt (g) after drying
Note	(1)	(1)	(2)
A	0.9719	1.4650	1.3717
B	0.8925	1.3485	1.2580
C	0.8480	1.2872	1.2087
D	0.8958	1.3525	1.2625

Notes: (1) Samples (CS-1) were conditioned to constant weight over LiCl saturated solution at 25.0 °C (RH 11.3 percent[7,8]) before and after reaction; standard uncertainty ≈ 0.0010 g. (2) Dry weights of reacted samples were measured after conditioning to constant weight over freshly-regenerated molecular sieve 4A at 25 °C (RH $<$ 0.1 percent[9]); standard uncertainty ≈ 0.0003 g.

2:1 phyllosilicate mineral willemseite $Ni_3Si_4O_{10}(OH)_2$[11] and on the nickel hydroxide polymorph β-$Ni(OH)_2$.[12]

The X-ray absorption near-edge structure (XANES) of the reaction samples (Figure 4a of the main paper) shows an uneven split white line, and two features at ≈ 8.365 keV and ≈ 8.395 keV (dashed vertical lines), distinctly different from the Ni^{2+}(aq) standard. The white line of the β-$Ni(OH)_2$ XANES standard shows a more even split peak compared to the sample spectra and the two features at ≈ 8.365 and ≈ 8.395 keV are shifted to higher energies in the reacted samples relative to the $Ni(OH)_2$ standard. These differences confirm that $Ni(OH)_2$ does not form in the samples. We reported similar observations on Co-S-H,[1] which Ni-S-H clearly resembles.

The EXAFS fits are shown as dashed black lines in Figure 4b, c of the main paper. Full details are given here in Table S4. The EXAFS spectra are fitted using the structure of willemseite.[11] These fits include three distinct scattering paths which all improved the respective fits, a Ni−O scattering path with a coordination number [CN] of 6 at 2.058-2.063 Å, a Ni−Ni scattering path with CN 6 at 3.093-3.110 Å, and a Ni-Si scattering path with CN

Table S4 Summary of the fits for the Ni-S-H samples using shell-by-shell analysis. Listed are the R-factor for the best fit, the amplitude correction factor (s_0^2), the energy shift ΔE_0, the coordination number C.N., radial distance R, the Debye-Waller factor σ^2 of the respective scattering paths, and the F-test statistic (statistical probability that the respective scattering path improves the fit).[13] In parentheses are the uncertainties of the last digit(s) calculated by ARTEMIS.[14] Asterisks (*) denote values that were fixed during fitting.

Sample	R-factor	s_0^2	ΔE_0	Path	C.N.	R	σ^2	F-test
			eV			Å	Å^{-2}	percent
Ni^{2+}	0.0032	0.85(3)	−2.2(4)	Ni−O	6*	2.055(3)	0.0046(5)	–
β-$Ni(OH)_2$	0.0079	0.68(4)	−3.0(6)	Ni−O	6*	2.064(5)	0.0059(8)	–
				Ni−Ni	6*	3.121(4)	0.0057(5)	100.0
Ni/CS, 14 d								
0.339 *m*	0.0069	0.70(5)	−2.2(8)	Ni−O	6*	2.061(7)	0.0067(10)	–
				Ni−Ni	6*	3.093(6)	0.0072(7)	100.0
				Ni−Si	4*	3.257(28)	0.0088(46)	99.4
71 m*m*	0.0028	1.03(5)	−3.5(5)	Ni−O	6*	2.058(4)	0.0082(7)	–
				Ni−Ni	6*	3.110(9)	0.0092(9)	100.0
				Ni−Si	4*	3.243(16)	0.0066(29)	100.0
34 m*m*	0.0037	0.98(5)	−3.5(5)	Ni−O	6*	2.056(4)	0.0072(8)	–
				Ni−Ni	6*	3.108(11)	0.0082(9)	100.0
				Ni−Si	4*	3.231(18)	0.0062(33)	100.0
7.0 m*m*	0.0039	0.86(4)	−2.8(5)	Ni−O	6*	2.063(4)	0.0060(7)	–
				Ni−Ni	6*	3.108(9)	0.0080(8)	100.0
				Ni−Si	4*	3.246(17)	0.0073(36)	99.9

4 at 3.231–3.257 Å. These radial distances (Table 1 of main paper), are similar to the those extracted from crystallographic data on willemseite[11] and on an end-member 1:1 Ni-phyllosilicate.[15] This suggests that the coordination environments of Ni in Ni-S-H resembles a phyllosilicate, supporting the XRD and FTIR results. This Ni-S-H phase is similar to the Co-S-H phyllosilicate described previously.[1] XAS has been used elsewhere to identify Ni phyllosilicates formed by reaction of Ni sorbed on montmorillonite,[16,17] and formed hydrothermally via an amorphous precursor from alkaline metasilicate.[18]

S5 Sharp-front diffusion of Ni^{2+} into CS bars

In addition to the SF tests on CS packed beds described in the main paper, we have carried out SF tests using rectangular bars of CS cut from CS-1 material. When the end face of the bar is in contact with a $Ni(NO_3)_2$ solution, the replacement reaction occurs by diffusion of Ni^{2+} ions into the porous CS. The bars are prepared by saturating under vacuum with distilled water to suppress capillarity effects. Fig S3a shows that a well-defined reaction zone

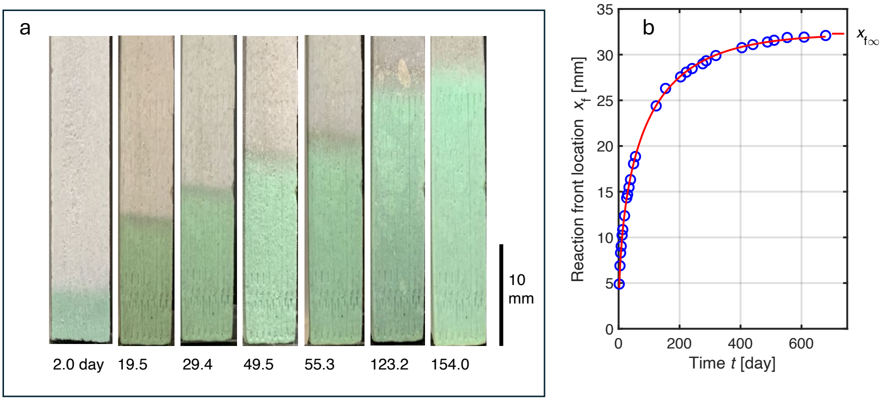

Figure S3 **a** Time-lapse images of a Ni^{2+}-sequestration front advancing into a rectangular CS bar, 60.0 mm long (part length shown), base face 9.36 × 8.63 mm^2; the base in contact with $Ni(NO_3)_2$ solution, $b(\mathrm{Ni})_0$ = 0.33 mol/kgw; bar pre-saturated with distilled water; test temperature 25.0 °C. **b** Position of reaction front vs time (blue circles), fit to SF model (red line), main paper Eqn (7); fit parameters α_0 = 5.812±0.167 day mm^{-1}, α_1 = 32.29±0.22 mm. Uncertainties ± 1 s.d.

develops and advances slowly along the bar. The advance of the reaction front is accurately represented by the SF model described in the main paper (Fig S3b). The transport parameter K calculated from the fit parameter α_0 has the value $(4.0 \pm 0.1) \times 10^{-10}$ m^2/s, in close agreement with the K values obtained from SF tests on packed beds and reported in the main paper.

Similar tests have been carried out on CS bars with Co^{2+} solutions. A full SF analysis will be published separately.

S6 Water-vapour sorption isotherms of CS and Ni-S-H

Water-vapour sorption isotherms of CS and Ni-S-H at 25°C are shown in Figure S4. Data on untreated CS were obtained using an Aquadyne DVS instrument, using small blocks of intact CS-1 material[1]; sorption data on Ni-S-H were obtained by accurate weight measurements on small blocks fully reacted by immersion in $Ni(NO_3)_2$ solution (0.33 mol/kgw) and conditioned over molecular sieve 4A desiccant (Brownell) and a series of saturated salt solutions. In Figure S4, fractional mass gain F_m is plotted against water activity $a_\mathrm{m} = p_\mathrm{w}/p_\mathrm{w0}$, where p_w is the water vapour pressure and p_w0 the saturated water vapour pressure at the measurement temperature. We note that the

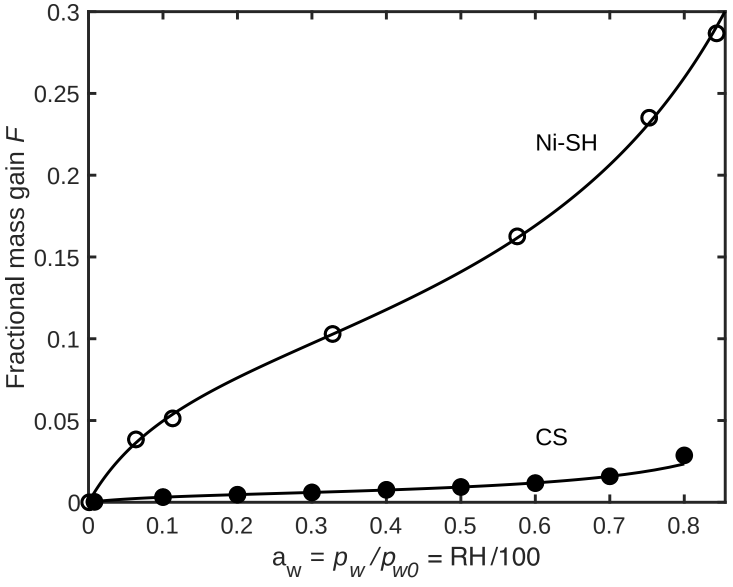

Figure S4 Water-vapour sorption isotherms of CS and Ni-S-H measured at 25 °C. The variation of fractional mass gain F with water vapour activity $a_w =$ RH/100 is fitted (solid lines) to the Guggenheim-Anderson-De Boer (GAB) isotherm function $F = F_m c_G k_G a_w / \{(1 - k_G a_w)[1 + (c_G - 1) k_G a_w]\}$, where F_m is the fractional mass gain at mono-layer coverage and c_G, k_G are the GAB parameters (CS: F_m (5.22 ± 0.35) × 10^{-3}, c_G 10.66 ± 2.68, k_G 0.979 ± 0.029; Ni-S-H: F_m (9.54 ± 0.42) × 10^{-2}, c_G 10.38 ± 2.46, k_G 0.813 ± 0.011.) Apparent GAB water-vapour specific surface areas are: CS, 19.4 ± 1.3 $m^2\,g^{-1}$; Ni-S-H, 355 ± 15 $m^2\,g^{-1}$. Ni-S-H data obtained from five replicate blocks; figure shows mean adsorption-branch isotherm.

nominal surface area a_s estimated from the GAB isotherm is $355 \pm 15\,\mathrm{m}^2/\mathrm{g}^{-1}$, about 30 percent higher than found previously for Co-S-H[1] using the same CS-1 starting material.

S7 Ni sequestration rate: dependence on weight of CS used

In our analysis of Ni sequestration kinetics in the main paper it is assumed that the rate of sequestration (mol/h) was proportional to the amount of CS used. This assumption, although reasonable, was not tested in the stirred batch experiments reported in the main paper. In these experiments, a fixed amount of CS (1.00 g) was used in all runs.

In order to test the assumption, an additional series of stirred-batch tests was carried out in which the mass of CS used, m_{CS}, was varied from 0.10 g to 1.80 g, all CS taken from the same batch at constant particle size. The quantity of nickel nitrate solution was fixed at either 25.00 g or 12.00 g. The concentration of the solution was adjusted to ensure that in all runs there was sufficient CS to achieve complete removal of Ni from solution. The amount of Ni initially present in solution is denoted n_{Ni}, as in Eqn 3 of the main paper. In these tests, the clearance time for Ni removal, t_c, was determined as the time at which the supernatant solution became colourless. Replicate runs, supported by dimethylglyoxime colour tests for Ni, show that t_c can be estimated with an uncertainty of at most 5 percent. In Fig S5 the quantity n_{Ni}/t_c is plotted against m_{CS}. If the sequestration rate is proportional to m_{CS}, then we expect that $n_{\mathrm{Ni}}/t_c = k m_{\mathrm{CS}}$, where k is the sequestration rate constant. The data show that the relation is linear over an eighteen-fold variation of m_{CS}. The slope of this line provides an estimate of k. The value of 0.16 mol/(kg CS · h) can be compared with the value of 0.27 mol/(kg CS · h) given in the main paper from earlier experiments. The values differ because the CS used in these tests

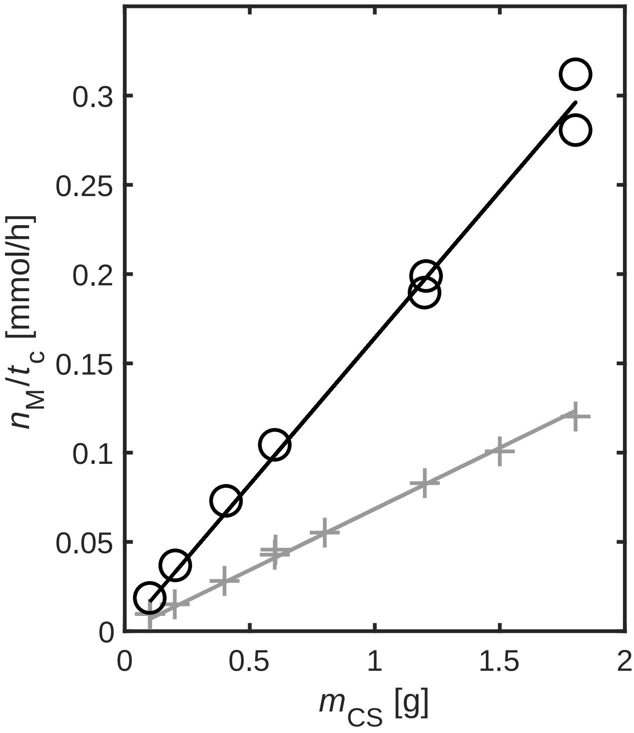

Figure S5 Rate of sequestration of Ni by CS: variation with initial weight of CS m_{CS}. Initial amount of Ni n_M, M = Ni, clearance time t_c (black circles); black line is linear fit through the origin with slope 0.164 mmol/(g CS · h). The grey symbols (+) and line show the corresponding data for Co^{2+} [1] (initial weight of Co n_M, M = Co). All measurements at 25 °C.

came from the Hamstad batch (CS-1) and has a larger particle size than CS-2. In these tests the value of k for Ni sequestration is about 2.4 times greater than the value previously reported[1] for Co at the same temperature, but without control of particle size (and hence surface area).

S8 Water absorption properties: CS as sponge

Dry CS absorbs water both rapidly and in large quantities. The standard properties to characterise the ability of a porous material to soak up water by capillarity are the sorptivity S and the fractional Hirschwald coefficient

h.[19] CS has exceptionally high values of both.[2,19,20] The measured S is 9.5±1.0 mm/min$^{1/2}$, and the measured h 0.81–0.90.

These values mean that 1 m^3 of CS absorbs about 0.75 m^3 of water. Since 1 m^3 of CS-2 weighs 185 kg, it soaks up more than four times its own weight of water. In the form of a slab 100 mm thick (a standard CS product dimension), CS on immersion takes up its total absorption capacity in about 15 min.

S9 Reaction of CS with $NiSO_4$ aqueous solution

Most of the tests reported in the main paper were carried out with aqueous solutions of nickel(II) nitrate, $Ni(NO_3)_2$. Here we mention some observations of the reaction of CS with solutions of nickel(II) sulfate, $NiSO_4$. When CS reacts with nickel nitrate, Ca^{2+} ions are released into solution in the Ni-S-H replacement reaction. Since $Ca(NO_3)_2$ is highly soluble, all Ca^{2+} remains in solution. However when CS reacts with nickel sulfate, the external solution may easily become supersaturated with respect to the sparingly soluble mineral gypsum $CaSO_4 \cdot 2\,H_2O$ (solubility 2.08 g $CaSO_4$/kgw at 25 °C). Figure S6 shows that when a CS core is placed in contact with a nickel sulfate solution, gypsum crystallites with distinctive stellate morphology[21,22] are formed on the surface of the core. The formation of gypsum provides additional evidence of the release of Ca^{2+} in the CS/Ni-S-H replacement reaction.

S10 Solution densities

Densities of nickel nitrate solutions calculated from[23] are given in Table S5.

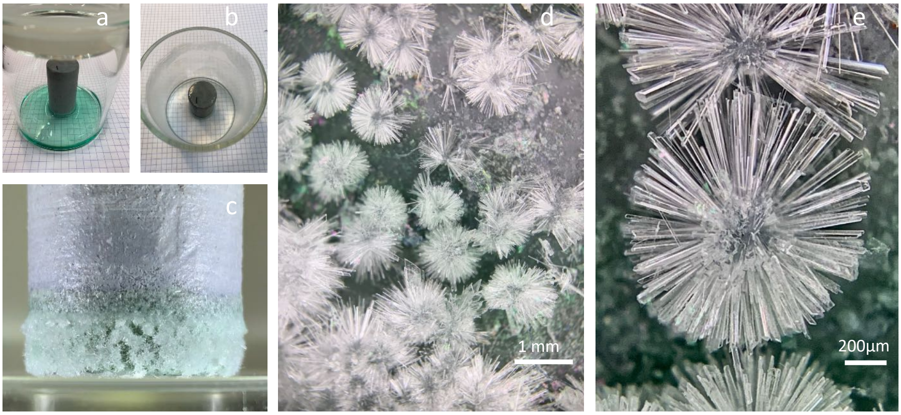

Figure S6 (a) A CS core (25 mm dia), pre-saturated with distilled water, placed in contact with a green $NiSO_4$ stock solution, 0.322 mol/kgw, at 25 °C; (b) colourless solution showing complete clearance of Ni from solution after 21 days; (c) crust of crystalline material on base and sides of the core in contact with solution shows stellate/spherulitic gypsum morphology [21,22]; (d, e) individual spherulites ≈ 100–500 μm dia.

Table S5 Aqueous $Ni(NO_3)_2$ solutions at 25 °C: Interconversion of molality *b*, amount concentration *c*, and solution density ρ_{soln}

Molality b(Ni) mol kgw^{-1} *m*	Amount concentration c(Ni) mol L^{-1}	Density ρ_{soln} kg m^{-3}
0.010	0.010	998.6
0.020	0.020	1000.1
0.030	0.030	1001.7
0.040	0.040	1003.2
0.050	0.050	1004.7
0.100	0.099	1012.3
0.150	0.149	1019.8
0.200	0.198	1027.2
0.250	0.247	1034.5
0.300	0.296	1041.8
0.400	0.394	1056.2
0.500	0.490	1070.3
0.600	0.586	1084.3
0.700	0.681	1098.1
0.800	0.776	1111.6
0.900	0.870	1125.1
1.000	0.962	1138.3

Note: Amount concentration $c = b\rho_{soln}/(1+bM)$ where the molar mass of $Ni(NO_3)_2$ M = 0.18270 kg/mol.

References

[1] A. Hamilton, P. Bots, H. Zhou, B. Liu and C. Hall, *Scientific Reports*, **2024**, *14*, 7052.

[2] S. Roels, J. Carmeliet, H. Hens, O. Adan, H. Brocken, R. Cerny, Z. Pavlik, C. Hall, K. Kumaran, L. Pel and R. Plagge, *Journal of Thermal Envelope and Building Science*, **2004**, *27*, 307–325.

[3] A. Hamilton and C. Hall, *Journal of Building Physics*, **2005**, *29*, 9–19.

[4] C. T. Do, D. P. Bentz and P. E. Stutzman, *Journal of Building Physics*, **2007**, *31*, 55–67.

[5] A. Hamilton and C. Hall, *Journal of Building Physics*, **2007**, *31*, 69–71.

[6] C. Hall, G. J. Lo and A. Hamilton, *Measurement Science and Technology*, **2022**, *34*, 027004.

[7] C. Hall, G. J. Lo and A. Hamilton, *Materials and Structures*, **2024**, *57*, 39.

[8] L. Greenspan, *Journal of Research of the National Bureau of Standards. Section A, Physics and Chemistry*, **1977**, *81*, 89–96.

[9] A. Gorbach, M. Stegmaier and G. Eigenberger, *Adsorption*, **2004**, *10*, 29–46.

[10] B. Ravel and J. J. Rehr, *Journal de Physique IV*, **1997**, *7*, 229–230.

[11] S. De Waal, *American Mineralogist: Journal of Earth and Planetary Materials*, **1970**, *55*, 31–42.

[12] V. Y. Kazimirov, M. Smirnov, L. Bourgeois, L. Guerlou-Demourgues, L. Servant, A. Balagurov, I. Natkaniec, N. Khasanova and E. Antipov, *Solid State Ionics*, **2010**, *181*, 1764–1770.

[13] L. Downward, C. Booth, W. Lukens and F. Bridges, *AIP Conference Proceedings*, **2007**, pp. 129–131.

[14] B. Ravel and M. Newville, *Journal of Synchrotron Radiation*, **2005**, *12*, 537–541.

[15] A. Levin, E. Khrapova, D. Kozlov, A. Krasilin and V. Gusarov, *Journal of Applied Crystallography*, **2022**, *55*, 484–502.

[16] R. Dähn, A. Scheidegger, A. Manceau, M. L. Schlegel, B. Baeyens, M. H. Bradbury and M. Morales, *Geochimica et Cosmochimica Acta*, **2002**, *67*, 1935–1935.

[17] X. Tan, J. Hu, G. Montavon and X. Wang, *Dalton Transactions*, **2011**, *40*, 10953–10960.

[18] A. Dumas, M. Mizrahi, F. Martin and F. G. Requejo, *Crystal Growth & Design*, **2015**, *15*, 5451–5463.

[19] C. Hall and W. D. Hoff, *Water transport in brick, stone and concrete*, CRC Press, London and New York, 3rd edn, **2021**.

[20] I. Ioannou, C. Charalambous and C. Hall, *Materials and Structures*, **2017**, *50*, 1–12.

[21] A. G. Reiss, J. Ganor and I. Gavrieli, *Crystal Growth & Design*, **2019**, *19*, 6954–6962.

[22] C. Jia, G. Zhu, B. A. Legg, B. Guan and J. J. De Yoreo, *Crystal Growth & Design*, **2022**, *22*, 6582–6587.

[23] B. S. Krumgalz, R. Pogorelsky and K. S. Pitzer, *Journal of Physical and Chemical Reference Data*, **1996**, *25*, 663–689.
